# aiSysMet: AI-powered systems metabolomics for biomarker discovery

**DOI:** 10.1093/bioinformatics/btag520

**Published:** 2026-07-15

**Authors:** Habtom Ressom, Linge Yan, Hongyu Ao, Xinran Zhang, Sara Hashemi, Rency Varghese, Bardia Nezami, Dawit Mengistu

**Affiliations:** OmicsCraft, Washington, DC, United States; OmicsCraft, Washington, DC, United States; OmicsCraft, Washington, DC, United States; OmicsCraft, Washington, DC, United States; OmicsCraft, Washington, DC, United States; OmicsCraft, Washington, DC, United States; OmicsCraft, Washington, DC, United States; OmicsCraft, Washington, DC, United States

## Abstract

**Motivation:**

Metabolomics plays an essential role in the growing systems biology approaches to unravel the relationships between metabolites and diseases. Liquid chromatography–mass spectrometry (LC-MS) is central to this effort because it can profile many metabolites from limited material. Yet, in a typical untargeted LC-MS-based metabolomics study, the majority of detected peaks remain unannotated, largely due to incomplete spectral libraries and uncertainties in peak picking, alignment, and the handling of isotopes and adducts. These limitations hinder seamless integration with other omics layers.

**Results:**

We developed an AI-powered platform (aiSysMet) that uses statistical, machine learning, and deep learning methods for metabolomics data processing, metabolite annotation, and integrative analysis of multi-omics data. The platform’s interactive and modular web interface allows users to easily build data analysis pipelines that can be executed in the cloud.

**Availability:**

aiSysMet is freely available for non-commercial users on https://tools.omicscraft.com/aiSysMet.

## 1 Introduction

Despite a large accumulation of metabolomics data acquired over the past several years, effective use of these data for biomarker discovery has been very limited. This is in part due to the lack of tools that: (1) accurately determine the identity of disease-associated analytes, thus limiting the ability to perform subsequent biomarker validation; (2) integrate metabolomics with other multi-omics data to evaluate the relationship between metabolites and diseases at the systems level; and (3) offer an all-in-one solution to directly engage experimentalists and computational scientists in metabolomics data analysis without the need to develop their own codes or learn disparate tools.

Various software platforms have been developed to address the above challenges. For example, MetaboAnalyst is commonly used for downstream statistics, biomarker discovery, and pathway-level analysis (Pang *et al.* 2024). XCMS is a foundational open-source framework (R/Bioconductor ecosystem) for preprocessing mass spectrometry data for peak detection, nonlinear retention-time correction, feature alignment, and peak matching to support untargeted metabolite profiling (Smith *et al.* 2006, Tautenhahn *et al.* 2012). The CAMERA package is frequently used with XCMS outputs to group related ions and annotate isotopes/adducts (Kuhl *et al.* 2012). MZmine is an open-source, modular platform for MS data processing, visualization, and molecular feature extraction (Pluskal *et al.* 2010). MS-DIAL emphasizes MS/MS-driven deconvolution and annotation, particularly in lipidomics; OpenMS provides a highly modular and scalable framework for automated workflows (Sturm *et al.* 2008). GNPS enables community-based MS/MS molecular networking for compound annotation; and commercial tools such as Compound Discoverer and Progenesis QI offer integrated, vendor-optimized LC–MS processing environments. Together, these tools illustrate the spectrum of metabolomics software design from open, modular frameworks and annotation platforms to commercial solutions.

Despite these resources, there is a growing need for a data analysis platform that offers an all-in-one solution from raw data processing of LC-MS data to biomarker selection, overcoming inconsistencies such as data formatting mismatches due to the use of disparate tools for the intermediate steps. To this end, we developed aiSysMet, which is an AI-powered and scalable cloud-based platform that offers end-to-end solutions for metabolomics data processing, metabolite annotation, and integrative analysis of multi-omics data. Various statistical, machine learning, and deep learning models are developed and implemented in a modular format; users can assemble their choice of modules to build and run pipelines. The platform provides the opportunity not only to increase the number of annotated analytes in metabolomics studies but also to integrate metabolomics with other multi-omics data, thereby enhancing the involvement of metabolomics in systems biology research for diagnostic and prognostic biomarker discovery, as well as basic science research.

## 2 Methods

In this section, we describe software components including databases, management utilities, pipeline builder, and data analytics modules implemented in aiSysMet.

### 2.1 Databases

We built two databases (MetDB and SpectDB) by integrating data from a large number of sources including KEGG (Caspi *et al.* 2008), NIST (Chang *et al.* 2007), MoNA (Kind *et al*. 2018), HMDB (Wishart *et al.* 2018), LIPID MAPS (Sud *et al.* 2007), METLIN (Guijas *et al.* 2018), and MMCD (Cui *et al.* 2008). Since each source covers only a fraction of the metabolome, our integrated databases yield a more comprehensive coverage. The process of building these integrated databases involved several steps including creation of a data lake, ETL (extract, transport and load), normalization, and curation. As each database uses a different format, we filled the missing values on a consensus achieved based on chemical identifier information retrieved for the metabolites by cross-referencing multiple databases. MetDB consists of about 2M compounds with several curated molecular features including compound name, molecular formula, monoisotopic mass, InChIKey, SMILES, and molecular fingerprints. The development of MetDB involved creation of a data lake of 14.5M compounds from more 30 sources, from which 3.5M compounds were extracted through ETL. After normalization via information from PubChem, about 2M unique compounds were identified and annotated via extensive curation. For each compound in MetDB, we calculated a fingerprint, which is represented by a binary vector of more than 7K entries. Each entry indicates the presence or absence of known substructures or properties as calculated by tools such as OpenBabel and pyFingerprint based on predefined structure libraries including FP3, FP4, PubChem, MACCS, and Klekota-Roth (Klekota and Roth 2008, O’Boyle *et al.* 2008, Wang *et al.* 2009, Duhrkop *et al.* 2015). SpectDB consists of 7.1 M MS/MS spectra acquired from about 0.9 M compounds through experimental and in-silico methods. The development of SpectDB involved creation of a data lake of 7.1 M spectral from 20 sources. For most metabolites, more than one spectrum is acquired with various instrument types or settings such as collision energies to increase the transferability of the spectral library across different experiments. Following ETL and normalization, over 3.7 M experimental and 3.6 M in-silico MS/MS spectra representing over 88 K and 806 K unique compounds, respectively, were identified. Each spectrum is annotated with compound details as in MetDB as well as precursor mass-to-charge ratio (m/z), collision energy, ionization mode, etc.

### 2.2 Management utilities

The management utilities in aiSysMet enable users to create and manage their data analysis activities as unique projects and to maintain their source and processed data in a secured cloud storage. The Project Manager utility organizes data in the user space (uploaded data or pipeline generated outputs) on a project basis to support easy management and hassle-free retrieval. Also, it facilitates collaboration among researchers by allowing sharing of project pipelines, data, and results in a secured and transparent manner. The Data Manager utility provides a secured cloud storage space to users to upload their data. It also organizes results generated in multiple projects in separate folders.

### 2.3 Pipeline builder

aiSysMet allows users to build data analytics pipelines that can be saved as a project and can be retrieved into the pipeline canvas through the Project Manager. Users can drag modules from the left pane to the pipeline canvas by connecting the modules according to the desired workflow or sequence of analysis. Each module can be configured and run one at a time, or a pre-configured pipeline consisting of multiple modules can be run as a project at once. Users can modify pre-composed pipelines in case they want to evaluate results.

As omics data analysis can often take a long time, failures may be encountered at any stage of the workflow execution. The software includes a feature to save intermediate results in the designated user space to enable resumption of interrupted jobs rather than restart them all over. This saves a significant cost and time of the researcher as hours of work and data processing could be wasted otherwise. The saving of the intermediate data allows users to inspect the intermediate results and debug their workflow.

### 2.4 Data import modules

The Data Import modules enable uploading data from local/cloud storage or pre-specified databases. The Data Upload module allows users to upload from local or cloud storage spaces unprocessed metabolomics data for processing, processed metabolomics data for metabolite annotation, and processed multiomics data for biomarker discovery. Unprocessed metabolomics LC-MS/MS data in mzXML or mzML formats can be uploaded with a list of precursor m/z values and a sample metadata sheet consisting of subject/sample-level information such as age, sex, disease group, survival time, batch, etc., if available. The CRDC Data Retrieval module searches and retrieves preprocessed multi-omics data from the NCI Cancer Research Data Commons (CRDC) based on user specified search criteria such as primary site, cohort, omics data type, etc.

### 2.5 MetCraft modules for omics data processing

The MetCraft modules analyze unprocessed LC-MS/MS metabolomics data in mzML or mzXML formats or apply various data treatment methods to any processed omics data uploaded in .csv format. The Peak Detection module performs peak pricing, peak integration, and peak alignment. The Adduct/Isotope Recognition module facilitates subsequent metabolite annotation steps by pre-annotation, i.e. recognizing adducts and isotopes through peak clustering. The Data Filter module allows users to select a subset of features for subsequent analyses. Features are removed based on a user-specified threshold for a coefficient of variation across all selected subjects and a threshold for the percentage of missing values. The Outlier Screening module applies Principal Component Analysis (PCA) to visually determine outliers—samples that look different from the majority. The Missing Value Imputation module uses methods such as mean value, small integer value, half-minimum, and k-nearest neighbor (KNN) to impute missing values. The Normalization module provides various data normalization and scaling methods such as quantile normalization, median normalization, *z*-score scaling, min-max scaling, etc. The Batch Correction module uses empirical Bayes frameworks to adjust data from large-scale studies affected by running order or batch acquisition (Stein *et al.* 2015).

### 2.6 MetaboQuest modules for metabolite annotation

The MetaboQuest modules include spectral matching, mass-based search for metabolite annotation, and other modules that help organize and rank putative metabolite IDs. The Spectral Matching module searches for putative metabolite IDs by matching MS/MS spectra with those in SpectDB. Users can enter an MS/MS spectrum as a list of m/z-intensity pairs or upload the data in mzML, mzXML, or plain text format. The uploaded data may contain single or multiple MS/MS spectra for a batch search. Spectral matching mimics the manual metabolite verification procedure by comparing acquired MS/MS spectra against those in MS/MS spectral libraries. The Compound Fingerprint Prediction module uses a deep learning model for predicting molecular formulae and fingerprints based on the pattern of MS/MS spectra. The predicted formulae and fingerprints are then used to help rank putative metabolite IDs. The Mass-Based Search module enables search for putative metabolite IDs in MetDB based on m/z values. Users can enter m/z values or use uploaded or processed data from a preceding module to search for putative IDs. Monoisotopic mass values calculated based on the m/z values and user-specified adducts, ionization mode, and mass tolerance in ppm are used for mass-based search. The IF-THEN Rule module allows users to select IF-THEN rules in order to combine, remove, or mark putative metabolite IDs. The Isotopic Pattern Analysis module assigns scores to putative metabolite IDs based on the similarity between the measured and theoretical isotopic patterns of their molecular formulae.

### 2.7 IntSys modules for multi-omics data integration

The IntSys modules allow users to identify significantly altered omics features. Each module can be used for analysis of single or multi-omics data. Multi-omics data are concatenated upon loading each omics data separately via the Data Upload module. Alternatively, the Data Integrator module can be used to integrate outputs from multiple modules at any stage into a consolidated data matrix for downstream analysis. The Univariate Statistical Analysis module analyzes preprocessed omics data using parametric (Student *t*-test) or non-parametric (Mann-Whitney *U*-test) statistical methods to identify significantly altered features between two independent groups of samples. It can also be used to analyze matched/paired samples (e.g. tumor and adjacent non-tumors) using parametric (paired *t*-test) or non-parametric (Wilcoxon signed-rank test) to identify significantly altered features between two independent groups of samples. Also, a univariate Cox proportional hazards model can be used to evaluate features for survival analysis. The Multivariate Regression Analysis module allows users to apply multivariate analysis including logistic regression, partial least squares discriminant analysis (PLS-DA), and Cox regression in conjunction with LASSO, Elastic Net, variable importance in projection (VIP), etc. to rank features associated with disease status or survival. The Network-Based Analysis module uses differential networks to compare correlations between analyte pairs in the disease group with those in the control group. The module helps recognize changes in pairwise interactions of analytes related to a disease. The Machine Learning module uses methods including support vector machine, random forest, and transformer-based deep learning models in conjunction with recursive feature elimination (RFE), VIP, LASSO, SHapley Additive exPlanations (SHAP), integrated gradient (IG) to rank disease-associated features. The module includes Random Survival Forest, Survival SVM, XGBoost, and transformer-based deep learning methods along with VIP, LASSO, SHAP, and IG to rank features associated with survival. The Multivariate and Machine Learning modules use cross-validation, bootstrapping, and hold-out approaches to evaluate feature ranking and classification or survival analysis models. Visualization tools include ROC curves, box plots, volcano plots, heatmaps, Kaplan–Meier plots, etc.

### 2.8 AI assistant

The AI Assistant is a RAG-powered feature that provides answers to user questions related to aiSysMet by referencing its system documentation and user manual as an internal knowledge base. The information from these sources is converted into embeddings and stored in a vector database. Users can query the AI Assistant questions such as steps to build a pipeline, configure a certain module, view results, etc., in plain English by typing and submitting their question using the prompt box provided. The RAG model searches in the vector database for a contextual answer to the query and generates a human-like response using an LLM. It is important to note that because the AI Assistant response is not generic information produced by the LLM, it does not provide satisfactory answers for questions outside the scope and context of our software.

## 3 Results

**Figure 1.**
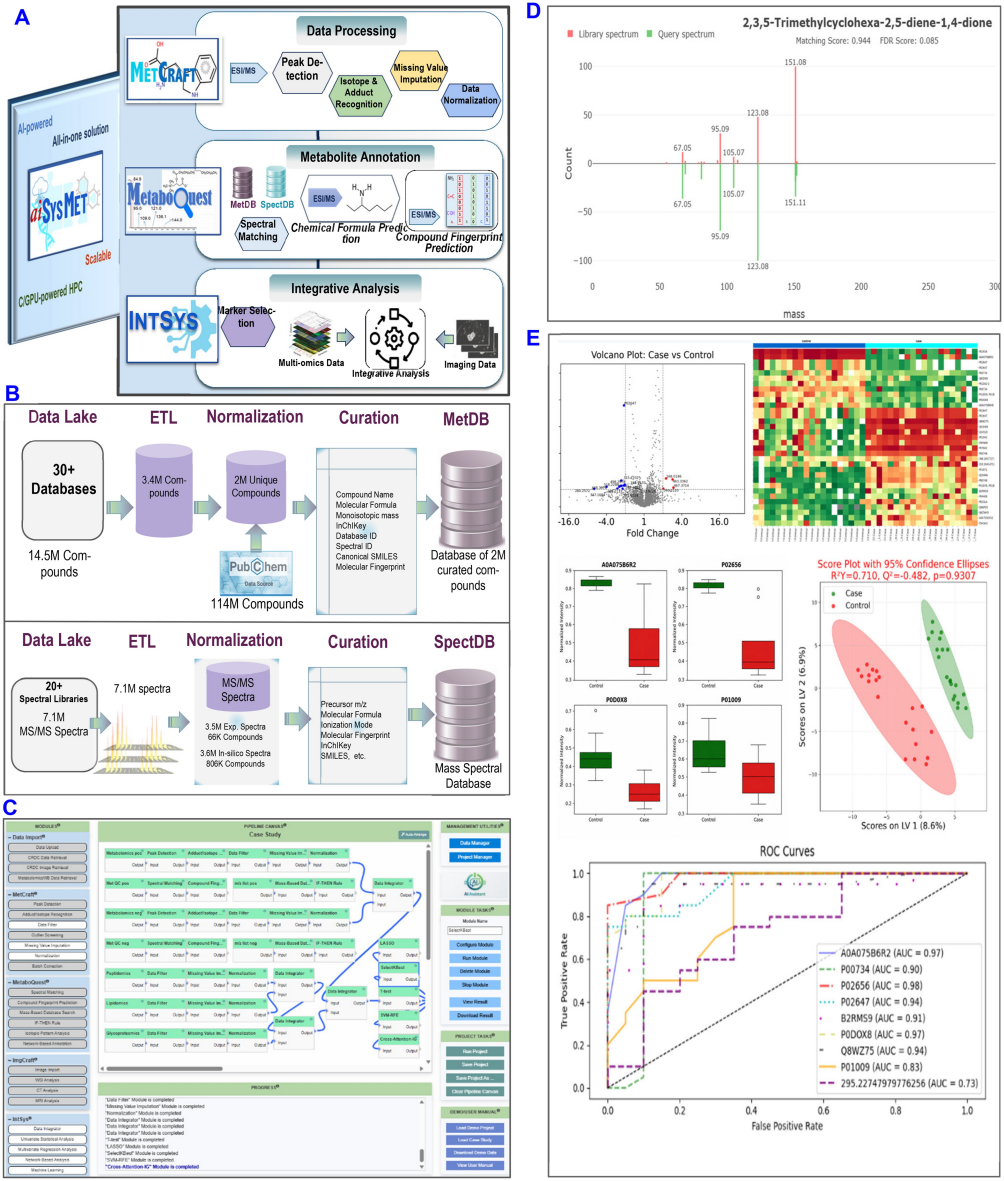
(A) Workflow and major functions of aiSysMet. (B) MetDB—a database of about 2M compounds included in aiSysMet for mass-based annotation. SpectDB—a database of 7.1M experimental and in-silico spectra representing about 0.9M metabolites included in aiSysMet for annotation based on spectral matching. (C) Screenshot of aiSysMet and pipelines built for a case study involving unprocessed metabolomic and processed multi-omics data. (D) Example of spectral matching result obtained by aiSysMet. (E) Analysis results obtained by integrative analysis of multi-omics data for biomarker discovery.

aiSysMet is a cloud-based platform that applies AI-powered analytics to support integrative analysis of multi-omics datasets, with a focus on metabolomics-driven biomarker discovery. It is accompanied by demo data that are accessible via the Data Manager utility to evaluate its performance in omics data processing, metabolite annotation, and multi-omics integration. In addition to evaluating its functionality using demo datasets and multi-omics data retrieved from the Cancer Research Data Commons (CRDC), aiSysMet was applied to the integrative analysis of multi-omics data from a case study focused on biomarker discovery. The case study included unprocessed metabolomics data in mzML format and processed multi-omics data (lipidomics, peptidomics, and glycoproteomics) acquired from 40 serum samples (20 cases and 20 controls) in a biomarker discovery study (Rashid *et al.* 2023, Varghese *et al.* 2025). The analysis pipeline and selected aiSysMet results are shown in Fig. 1C and E.

## 4 Conclusions and future plans

aiSysMet is a cloud-based, AI-powered platform that provides an end-to-end solution for raw metabolomics data analysis, metabolite annotation, and multi-omics integration to support diagnostic and prognostic biomarker discovery. The platform is developed in such a way that analysis can be performed by combining different modules and defining parameter settings. This flexibility is achieved by implementing a drag-and-drop pipeline builder that allows users to compose their pipelines according to the workflow they envisaged. It allows users to create and save multiple pipelines and analyze their data. Current limitations include the lack of built-in vendor raw-file conversion to mzML/mzXML, dependence on the coverage and quality of available MS/MS spectral libraries for metabolite annotation, limited support for user-defined custom MS/MS libraries, and potential memory overflow when very large datasets are analyzed using computationally intensive modules. Future development will focus on expanded spectral-library customization, improved handling of instrument-specific MS/MS variation, and quantitative benchmarking of runtime, memory usage, metabolite annotation accuracy, and scalability. Furthermore, future versions of aiSysMet will include improved scalability and a new category of modules (ImgCraft) for processing of multimodal medical images using deep learning methods such as vision transformers. Also, IntSys will support integration of multi-omics and imaging data for disease classification and survival analysis as well as identification of diagnostic and prognostic biomarkers.
